# Cinaciguat prevents the development of pathologic hypertrophy in a rat model of left ventricular pressure overload

**DOI:** 10.1038/srep37166

**Published:** 2016-11-17

**Authors:** Balázs Tamás Németh, Csaba Mátyás, Attila Oláh, Árpád Lux, László Hidi, Mihály Ruppert, Dalma Kellermayer, Gábor Kökény, Gábor Szabó, Béla Merkely, Tamás Radovits

**Affiliations:** 1 Heart and Vascular Center, Semmelweis University, Városmajor u. 68., 1122 Budapest, Hungary; 2 Institute of Pathophysiology, Semmelweis University, Nagyvárad tér 4., 1089 Budapest, Hungary; 3 Department of Cardiac Surgery, University of Heidelberg, Im Neuenheimer Feld 110., 69210 Heidelberg, Germany

## Abstract

Pathologic myocardial hypertrophy develops when the heart is chronically pressure-overloaded. Elevated intracellular cGMP-levels have been reported to prevent the development of pathologic myocardial hypertrophy, therefore we investigated the effects of chronic activation of the cGMP producing enzyme, soluble guanylate cyclase by Cinaciguat in a rat model of pressure overload-induced cardiac hypertrophy. Abdominal aortic banding (AAB) was used to evoke pressure overload-induced cardiac hypertrophy in male Wistar rats. Sham operated animals served as controls. Experimental and control groups were treated with 10 mg/kg/day Cinaciguat (Cin) or placebo (Co) p.o. for six weeks, respectively. Pathologic myocardial hypertrophy was present in the AABCo group following 6 weeks of pressure overload of the heart, evidenced by increased relative heart weight, average cardiomyocyte diameter, collagen content and apoptosis. Cinaciguat did not significantly alter blood pressure, but effectively attenuated all features of pathologic myocardial hypertrophy, and normalized functional changes, such as the increase in contractility following AAB. Our results demonstrate that chronic enhancement of cGMP signalling by pharmacological activation of sGC might be a novel therapeutic approach in the prevention of pathologic myocardial hypertrophy.

Long term presence of pathologic myocardial hypertrophy is a major underlying cause of heart failure (HF). One of its main inducing factors is pressure overload of the left ventricle (LV), which causes concentric LV hypertrophy (LVH) with collagen accumulation and subsequent impairment of diastolic function. This adverse remodelling of the LV can result in HF with preserved ejection fraction (HFpEF), a condition that is increasingly investigated, as it equals HF with reduced ejection fraction (HFrEF) both in outcomes and numbers^1^. The bulk of patients who develop HFpEF suffer from persistent hypertension^2^. It is well known that hypertensive heart disease (HHD) is initially characterized by compensated concentric LVH, which, eventually, transits to overt HF. Although effective pharmacological and device therapies have been developed to decrease the burden of HFrEF^3^, clinical trials targeting patients with HFpEF had neutral results to this date^1^,^3^. Therefore, new therapeutic approaches might be feasible in addressing the growing public health burden of HFpEF.

Cyclic GMP (cGMP) is an important regulator of many physiological and pathophysiological processes in the cardiovascular system, including cardiac remodelling^4^. Under physiological conditions, the major source of cGMP in cardiomyocytes is soluble guanylate cyclase (sGC), which is activated by nitric oxide (NO)^5^. The main effector of cGMP inside the cardiomyocyte is the cGMP-dependent protein kinase (PKG), which was identified as a key negative regulator of LVH and adverse remodelling^6^,^7^. Various cardiovascular diseases result in an impaired signalling through the NO-cGMP-PKG pathway^8^. It has previously been shown that elevated cytosolic levels of cGMP originated either from blockade of its degrading enzyme, phosphodiesterase type 5 (PDE-5)^9^ or from increasing its production by stimulating or activating sGC^10^,^11^ preserved myocardial structure and function in experimental ischemia-reperfusion models. Therefore, elevating myocardial cGMP levels might prove to be an effective new approach of preventing the development of pathologic LVH.

A new group of drugs named sGC activators has been developed^12^ in order to counteract the impairment of the NO-cGMP-PKG pathway. Cinaciguat (BAY 58-2667) is the firstly characterized and most potent member of the sGC activators^13^. It can activate sGC independently of its haem moiety, which serves as the physiological NO sensor in sGC^13^. Under pathologic conditions associated with increased nitro-oxidative stress (such as diabetes, ischaemia/reperfusion or LVH^8^), the haem of sGC becomes oxidized, which renders it incapable of binding NO and facilitates its dissociation from the enzyme^14^, resulting in the inability of sGC to generate cGMP. Cinaciguat activates these inactive forms of sGC more potently than it does the reduced sGC^14^. This potentially disease-selective mode of action makes activators of sGC especially tempting new tools in our pharmacological therapeutic inventory.

In our present study, we aimed at characterizing the cardiac effects of Cinaciguat in a rat model of pressure overload-induced LVH. We used abdominal aortic banding (AAB) to induce pressure overload in our animals, which is a well-established and widely used procedure to evoke hypertension and pathologic LVH in rodents^15^,^16^,^17^.

## Results

### Echocardiography

**Figure 1:**
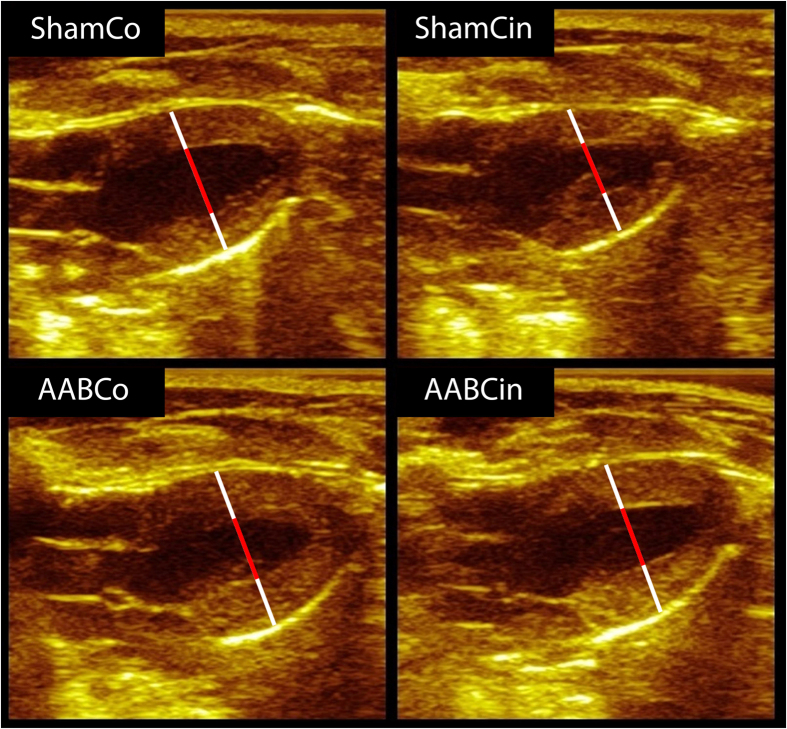
Representative echocardiographic images from the 6^th^ week in diastole. White bars represent walls, and red bars show cavities. Note the difference among groups in the length of the bars.

The echocardiographic measurement performed on the 3^rd^ postoperative week verified significantly elevated LV wall thickness values, relative wall thickness (RWT) and estimated LV mass (LVM) in the AABCo group compared to ShamCo without significant changes in chamber dimensions (Table 1). LVH increased over the second half of the treatment period in the AABCo animals (Table 1, Fig. 1), which was accompanied by significantly elevated LV end-systolic (LVESD) diameter compared to ShamCo. The Cinaciguat treatment in aortic banded rats resulted in significantly decreased LV diastolic wall thicknesses, LVM and LVM index (LVMi) compared to AABCo at both time points (Table 1). Systolic posterior wall thickness at the 6^th^ week of the treatment was also significantly decreased in the AABCin animals compared to the AABCo group, while ejection fraction (EF) and fractional shortening (FS) remained unchanged during the whole study (Table 1).

### Body and organ weights

There was no significant difference among the groups in body weight (Supplementary Table 3). Heart weight normalized to tibial length (HW/TL) was significantly higher in the AABCo rats than in the ShamCo or ShamCin animals (Supplementary Table 3, 29.3 ± 0.8 mg/mm ShamCo, 28.1 ± 0.9 mg/mm ShamCin vs. 38.4 ± 1.5 mg/mm AABCo, p < 0.05). HW/TL was significantly reduced in the AABCin animals compared to the AABCo rats (33.5 ± 0.7 mg/mm AABCin, p < 0.05). Relative wet lung (LuW/TL) weight was significantly increased in the AABCo group compared to ShamCo. This parameter did not differ from ShamCo in the AABCin animals (Supplementary Table 3).

### Hemodynamic measurements

Basic hemodynamic parameters, such as heart rate (HR), EF, stroke volume (SV), cardiac output (CO), or LV end-systolic volume (LVESV), and also parameters of preload, such as LV end-diastolic volume (LVEDV) and -pressure (LVEDP) were not significantly different among the groups (Table 2).

LV systolic (LVESP) and mean arterial blood pressure (MAP) proximal to the site of stenosis were significantly higher in both AAB groups than in the Sham groups, and neither of these parameters were affected by Cinaciguat (Table 2).

**Figure 2:**
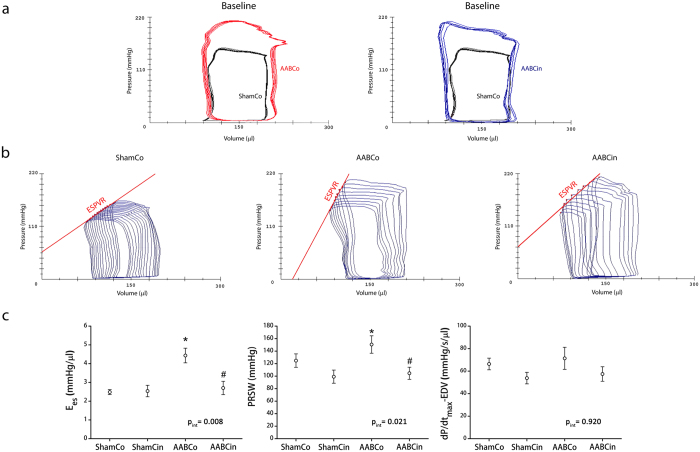
Cinaciguat normalises increased contractility in pressure overload. Baseline characteristics of pressure-volume relations did not differ in the AABCo and AABCin groups (**a**). P-V loops recorded during occlusion of the inferior caval vein (**b**) and the load independent indices of contractility derived from these measurements (**c**) show, however, that Cinaciguat significantly decreased the increase in contractility following abdominal aortic banding. PRSW: preload recruitable stroke work; E_es_: end systolic elastance; dP/dt_max_-EDV: maximum rate of pressure change – end diastolic volume relationship; p_int_: interaction *p* value *p < 0.05 vs. ShamCo; ^#^p < 0.05 vs. AABCo.

Maximum rate of LV pressure increment (dP/dt_max_) was significantly higher in AABCo animals than in ShamCo rats (Table 2). Load independent indices of contractility, such as end-systolic elastance (E_es_) and preload recruitable stroke work (PRSW) (Fig. 2), also showed that AABCo animals had significantly elevated LV contractility compared to ShamCo. These parameters, however, indicated a significant decrease of contractility in AABCin compared to AABCo rats. dP/dt_max_-end-diastolic volume relationship (dP/dt_max_-EDV) had a similar trend (Fig. 2).

Active relaxation was impaired in the AABCo rats compared to ShamCo, as evidenced by the time constant of LV pressure decay (τ), while it was similar to ShamCo in the AABCin animals (Table 2).

### Histology, immunohistochemistry, biochemistry

**Figure 3:**
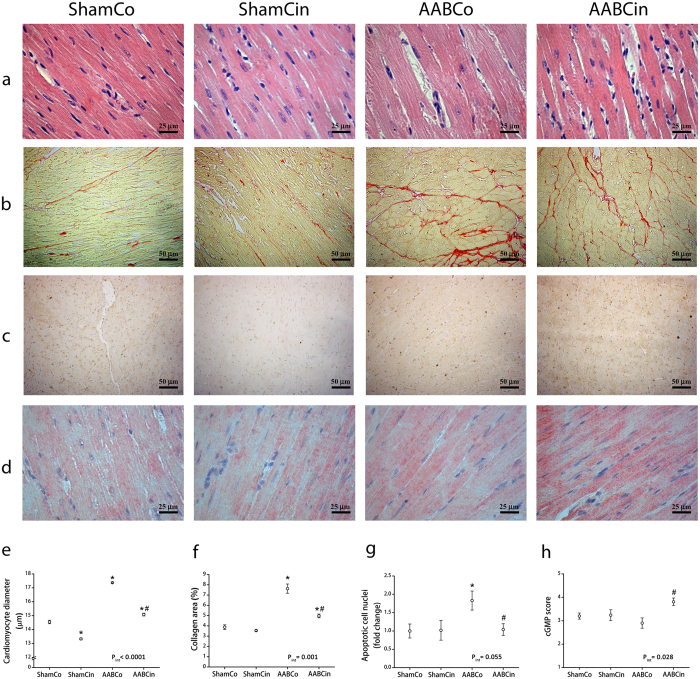
Histological alterations in pressure overload are blunted by Cinaciguat. Differences between the groups are illustrated in representative photomicrographs of left ventricular (LV) myocardial sections with haematoxylin-eosin (**a**) and Picrosirius red staining (**b**), TUNEL (**c**) and cGMP immunohistochemistry (**d**). Average cardiomyocyte diameter (**e**) and collagen area of subendocardial LV myocardium (**f**) was significantly elevated in the AABCo group compared to ShamCo, both of which alterations were significantly decreased following Cinaciguat treatment. TUNEL staining revealed a significant increase in the number of apoptotic cell nuclei in the AABCo group compared to ShamCo and AABCin (**g**). cGMP score (**h**) was significantly higher in the AABCin group than in the AABCo animals. p_int_: interaction *p* value *p < 0.05 vs. ShamCo; ^#^p < 0.05 vs. AABCo.

Morphological changes were present on the microscopic level as well. Average cardiomyocyte width was significantly increased in the AABCo group compared to ShamCo (Fig. 3a and e), which was significantly lower in AABCin rats than in AABCo animals. Quantitative analysis of heart sections stained with Picrosirius red showed that the collagen area of subendocardial LV myocardium was significantly increased in the AABCo group compared to ShamCo, which was significantly decreased in the AABCin rats compared to AABCo (Fig. 3b and f). Terminal deoxynucleotidyl transferase dUTP nick end labelling (TUNEL) revealed a significant increase in the number of apoptotic cell nuclei in the AABCo group compared to ShamCo and AABCin (Fig. 3c and g).

**Figure 4:**
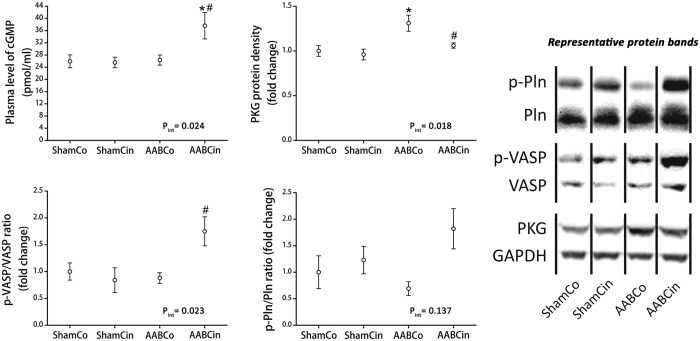
Effects of Cinaciguat on cGMP signalling in pressure overload. The strong sGC activating effect of Cinaciguat during pathologic conditions was confirmed by measuring plasma level of cGMP, which was significantly elevated in AABCin rats. cGMP activates PKG, which then phosphorylates VASP and Pln, phosphorylation ratio of which are widely used markers of PKG activity. Both of these were markedly increased despite the unchanged amount of PKG in the AABCin group, indicating increased PKG activity in these animals. Representative Western blot bands are shown for each group and investigated protein on the right. PKG: protein kinase G; Pln: phospholamban; VASP: vasodilator stimulated phosphoprotein; p_int_: interaction *p* value *p < 0.05 vs. ShamCo; ^#^p < 0.05 vs. AABCo.

Analysing immunohistochemical staining on myocardial sections for cGMP resulted in significantly higher score in AABCin rats than either in ShamCo or AABCo animals (Fig. 3d and h). Plasma level of cGMP was also significantly elevated in the AABCin animals compared to both ShamCo and AABCo groups (Fig. 4).

### mRNA analysis

**Figure 5:**
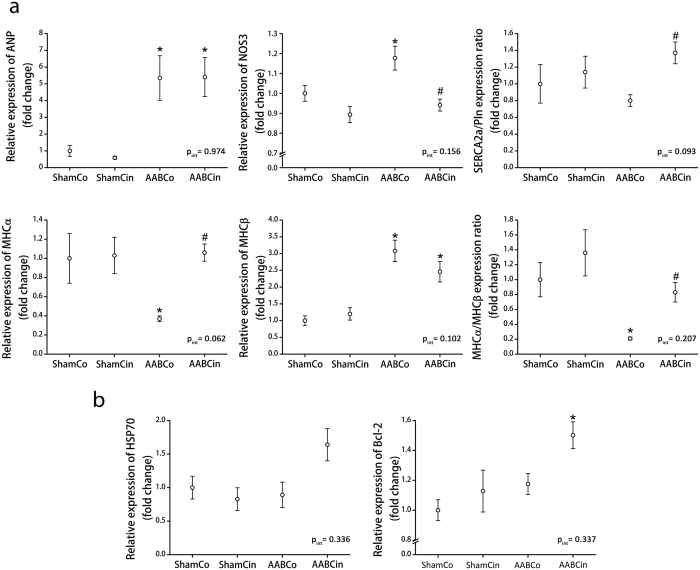
Gene expression changes are prevented in response to Cinaciguat treatment. (**a**) Aortic banding resulted in the reactivation of the foetal gene program, as evidenced by the elevated expression of ANP, MHCβ, and NOS3, the decreased expression of MHCα. Expression of NOS3 and MHCα along with the MHC isoform expression ratio was normalised by the Cinaciguat treatment following aortic banding. The SERCA2a/Pln expression ratio was significantly increased in the AABCin rats compared to the AABCo animals. (**b**) Both HSP70 and Bcl-2 expression was markedly elevated in the AABCin group, indicating reinforced anti-apoptotic signalling in these animals. ANP: atrial natriuretic peptide; Bcl-2: B-cell lymphoma 2; HSP70: 70 kDa heat shock protein; MHCα/β: α and β isoform of myosin heavy chain; NOS3: endothelial nitric oxide synthase; Pln: phospholamban; SERCA2a: sarcoplasmic and endoplasmic reticulum Ca^2+^ ATPase isoform 2a; p_int_: interaction *p* value *p < 0.05 vs. ShamCo; ^#^p < 0.05 vs. AABCo.

Pressure overload of the left ventricle resulted in elevated myocardial expression of atrial natriuretic peptide (ANP) and endothelial NO synthase (NOS3) (Fig. 5a), and decreased ratio of myosin heavy chain isoforms α and β (MHCα/MHCβ) expression (Fig. 5a), indicating the reactivation of the foetal gene program in the AABCo animals. The Cinaciguat treatment normalised the relative expression of NOS3 and the ratio of MHCα/MHCβ expression (Fig. 5a), while ANP expression was unaltered by the treatment (Fig. 5a). Expression ratio of sarco/endoplasmic reticulum Ca^2+^-ATPase 2a (SERCA2a) and phospholamban (Pln) was significantly elevated in the AABCin rats (Fig. 5a).

Anti-apoptotic signalling was reinforced by Cinaciguat, as evidenced by the significant increase in B-cell lymphoma 2 (Bcl-2) expression and the strong tendency towards higher expression of 70 kDa heat shock protein (HSP70) in the AABCin animals (Fig. 5b).

### Immunoblot analysis

Protein density of protein kinase G (PKG) was significantly elevated in myocardial homogenates of AABCo rats, while it was comparable to ShamCo in the AABCin group (Fig. 4). Phosphorylation ratio of vasodilator-stimulated phosphoprotein (VASP) and Pln are widely used indicators of PKG activity, both of which were elevated following the Cinaciguat treatment (Fig. 4).

## Discussion

In our current work we demonstrate for the first time that the chronic activation of sGC by Cinaciguat and the subsequent rise in cGMP levels efficiently reduce pressure overload-induced pathologic myocardial hypertrophy *in vivo* despite the unchanged loading of the LV. In parallel with the significant morphological changes, functional alterations were normalised by the Cinaciguat treatment following AAB.

*In vivo*, the major drive in the background of the hypertrophic response of cardiomyocytes to chronically increased afterload is the stretching of the cell membrane^18^,^19^. Recently published *in vitro* studies have shown that Cinaciguat has anti-hypertrophic effects in cultured neonatal rat cardiomyocytes^20^, suggesting that the chronic activation of the NO-cGMP-PKG pathway is capable of decreasing cardiomyocyte hypertrophy irrespective of the mechanical stress inflicted on cardiomyocytes by hemodynamic load. The significance of NO-cGMP-PKG signalling might be that it regulates a plethora of important mechanisms including Ca^2+^-related signalling pathways, troponin I^21^ and various ion channel phosphorylation^22^. Our present results are in line with the above mentioned anti-hypertrophic properties of sGC-activation. Furthermore, *in vivo* myocardial anti-hypertrophic effect of Cinaciguat in previously published works was suggested to be secondary to the amelioration of the primary disease (pulmonary hypertension^23^ and uraemia^24^) by the drug. In contrast, the primary disease in our model cannot be resolved by the drug, therefore we show here for the first time that Cinaciguat exerts a primary anti-hypertrophic effect *in vivo*. This effect is present irrespective of the hemodynamic loading of the LV, which might be the result of the increased activity of PKG due to the elevation of intracellular cGMP level by Cinaciguat. This is evidenced by myocardial and plasma cGMP-levels (Figs 3d,h and 4), and increased phosphorylation ratio of VASP and Pln (Fig. 4), both of which are widely used as markers of PKG activity^25^,^26^.

Although oxidation and thus inactivation of sGC has been reported in the development of LVH^27^, plasma cGMP levels were found to be unaltered in the AABCo group when compared to ShamCo. This finding might be explained by the overexpression of natriuretic peptides (such as ANP, Fig. 5a) and subsequent cGMP-production by particulate GC^28^, which could be interpreted as an ineffective compensatory reaction to sGC inactivation. Furthermore, pGC might not be able to directly replace the function of sGC in the cell; the different subcellular compartmentalisation of sGC versus pGC derived cGMP should be taken into account^29^.

A significant concentric LVH was present in the AABCo group by the 6^th^ week, as evidenced by RWT values. AWTd, PWTd and LVEDD were significantly decreased in the AABCin group compared to AABCo (Table 1). Indeed, LVMi estimated from our echocardiographic measurements showed that Cinaciguat significantly decreased the extent of LVH (Table 1). Our finding correlates with previous data about the PDE-5 inhibitor sildenafil, which also increases the amount of intracellular cGMP, and was shown to reduce LVH significantly^9^. Post mortem organ weight measurements correlated with these results: AABCo rats developed a significant increase both in absolute and relative heart weight compared to ShamCo, which is similar to previous data in this model^30^. This gain of heart weight was significantly decreased by the Cinaciguat treatment (Supplementary Table 3), which clearly reflects the anti-hypertrophic properties of Cinaciguat.

Chronically increased afterload induces compensatory remodelling of the myocardium. Unlike physiological myocardial hypertrophy that occurs in athletes, pathologic stimuli such as hypertension lead to maladaptive changes in the cellular structure of cardiomyocytes^31^. On the microscopic level, we found a significant increase in average cardiomyocyte width and subendocardial collagen area in the AABCo group compared to ShamCo (Fig. 3a,b,e and f). Treatment with Cinaciguat significantly reduced both average cardiomyocyte width and subendocardial collagen area in our aortic banded rats (Fig. 3a,b,e and f), which correlates well with the decrease observed in LVMi and heart weight (Table 1 and Supplementary Table 3) both in this study and with previous results^9^,^32^.

A major change in the subcellular phenotype characteristic to pathologic LVH is the reactivation of the foetal gene program^33^. Indeed, we observed a shift toward the expression of the less efficient, but less energy consuming β isoform of myosin heavy chain from the α isoform in the AABCo animals, a well-known change^34^ that was completely normalised by the Cinaciguat treatment, as observed in the AABCin group (Fig. 5a). This result is remarkable in the light of the similar loading of the LV, as MAP was comparable in the aortic banding groups (Table 2). It must be noted here that Cinaciguat has been critically discussed in recent publications due to its hypotensive effect in human clinical trials, which utilized the drug intravenously^35^,^36^. It is very important to emphasize, however, that consistently with other pharmacological agents, the pharmacokinetics of Cinaciguat is significantly different when administered orally. In line with this, according to previous reports, a single oral dose of 10 mg/kg Cinaciguat only mildly and transiently lowers blood pressure^13^,^37^. Furthermore, chronic oral administration of the drug in this dose did not significantly alter arterial blood pressure in the systemic circulation neither in murine models of pulmonary hypertension^23^ nor in a rat model of diabetic cardiomyopathy^38^. Similar results with oral Ataciguat and GSK2181236A, two further sGC activators have recently been reported in a rat myocardial infarction model and in spontaneously hypertensive stroke prone rats^10^,^39^. Conforming these data, we did not observe any changes in MAP of the rats in response to orally administered Cinaciguat at the time of the hemodynamic assessment, 24 h after the last application of the drug. Nevertheless, the observed robust overexpression of ANP in both AAB groups (Fig. 5a) provides evidence for unchanged loading, similar LV wall stretch and mechanical hypertrophic stimulus in the AABCo and AABCin animals.

Excessive stretching of the plasma membrane of cardiac myocytes could also induce programmed cell death^40^. Our results correspond with previous data, we observed a significant increase in the number of apoptotic cell nuclei in the AABCo group compared to ShamCo with TUNEL staining (Fig. 3c and g). This alteration was normalised by the Cinaciguat treatment, which improvement could be explained by reinforced anti-apoptotic signalling, as evidenced by the increased expression of Bcl-2 and HSP70 (Fig. 5b).

Thus, we observed a significant improvement of the detrimental changes occurring during pathologic LVH on all three observable (i.e., macroscopic, microscopic and molecular) levels in response to Cinaciguat treatment.

As described above, chronic overload of the LV results in pathologic morphological changes of the myocardium. These result in an initial, functionally compensated phase with hypertrophy and an increase in contractility, to compensate for the increased afterload. Eventually, however, decompensation with LV dilatation, systolic dysfunction and overt HF develops^41^. We found maintained systolic performance in our animals with echocardiography both on the 3^rd^ and 6^th^ week (Table 1), which suggests that our AABCo and AABCin animals were in the compensated hypertrophic phase throughout the experiment. The significantly increased LVESD in the AABCo animals, however, might anticipate LV dilatation and systolic dysfunction, while Cinaciguat effectively prevented this alteration as well (Table 1).

Analysis of P-V data acquired during invasive hemodynamic measurements provides more precise assessment of cardiac performance. E_es_ (the slope of ESPVR) was proposed as a fairly load-insensitive index of ventricular contractility. PRSW (the slope of the linear relation between SW and EDV) has been described as a parameter independent of chamber size and mass, and it is sensitive to contractile function of the ventricle^42^. These indices showed an increase in LV contractility in the AABCo group, which was not present following the Cinaciguat treatment (Table 2 and Fig. 2). These results are partially explained by the anti-hypertrophic effects of Cinaciguat, as described above and in previous studies^43^,^44^. Further important contributors to these results might be functional changes induced by the activation of PKG: inactivation of L-Type Ca^2+^-channels and activation of late rectifier K^+^-channels might both decrease intracellular Ca^2+^ concentration^22^. What is more, phosphorylation of troponin I by PKG could ameliorate Ca^2+^-sensitivity of cardiomyocytes^21^. Therefore, while not completely preventing adaptive compensatory hypertrophy (Table 1, Fig. 3a and e, Supplementary Table 3), Cinaciguat appears to attenuate the excess in the hypertrophic response that might not be required for the LV to withstand increased afterload^45^, in parallel with ameliorating all characteristic changes of pathological hypertrophy including fibrosis (Fig. 3b and f), apoptosis (Fig. 3c and g) and reactivation of the foetal gene program (Fig. 5a).

A hallmark of HHD is the impairment of LV diastolic function long before systolic dysfunction occurs, resulting clinically in the HFpEF phenotype. Both the decrease of passive compliance and impaired active relaxation of the LV can be in the background of diastolic dysfunction^46^. Despite the elevated subendocardial collagen area in the AABCo animals (Fig. 3b and f), LVEDP did not change compared to ShamCo (Table 2), which suggests that passive compliance of the LV was unaltered at this early stage of HHD. Increased collagen area in the AABCo group was present only in the subendocardial region, which correlates with previous results^32^. Collagen deposition, as observed by the same authors, expanded to the complete width of the LV wall when the duration of pressure overload was longer^32^. τ, on the other hand, which is the time constant of LV pressure decay and thus characterises active relaxation, was significantly increased in the AABCo group, suggesting impaired active relaxation (Table 2). There was no sign of diastolic dysfunction in the AABCin animals, which could be explained by the elevated ratio of expression of SERCA2a and Pln (Fig. 5a) and the increased phosphorylation ratio of Pln (Fig. 4) compared to AABCo. Both of these changes could contribute to facilitation of cytoplasmic Ca^2+^-clearance in the early phase of diastole, resulting in maintained active relaxation^47^,^48^.

Diastolic dysfunction causes backward failure initially in the pulmonary circulation^49^. In accordance with this, we found significantly elevated relative wet lung weight in our AABCo rats, while it was comparable to ShamCo in the AABCin animals (Supplementary Table 3).

### Limitations

LV geometry and the amount of fibrotic components in the myocardial wall might both influence LV contractility parameters measureable by PV-analysis. Despite this method being the current gold standard for assessing different aspects of cardiac function, it is not possible to separate these confounding factors from the true contractility of the myocardial sarcomere *in vivo*. Therefore, the observed increase in contractility in the AABCo group and conversely, normalization of contractility in AABCin animals might partially be caused by the structural differences of the LV wall between these groups.

## Conclusions

Our research group shows here for the first time that chronic activation of sGC by Cinaciguat prevents the development of pathologic myocardial hypertrophy *in vivo* irrespective of hemodynamic load. We observed the beneficial effect of sGC activation on morphological, functional and molecular levels as well. sGC activators therefore might prove to be an efficient new therapeutic approach in the treatment of pathologic myocardial hypertrophy.

## Materials and Methods

For more details, see the online supplementary material. All animals received humane care in compliance with the “Principles of Laboratory Animal Care”, formulated by the National Society for Medical Research and the Guide for the Care and Use of Laboratory Animals, prepared by the Institute of Laboratory Animal Resources and published by the National Institutes of Health (NIH Publication No. 85-23, Revised 1996). All procedures and handling of the animals during the study were reviewed and approved by the Ethical Committee of Hungary for Animal Experimentation. Young adult (10 weeks old, body weight = 220–240 g) male Wistar rats (n = 35) (“Toxi-Coop” Zrt., Dunakeszi, Hungary) were housed in a room with constant temperature of 22 ± 2 °C with a 12 h light-dark cycle, were fed a standard laboratory rat chow ad libitum and had free access to water.

### Abdominal aortic banding procedure

After acclimation, banding of the abdominal aorta (AAB, n = 19) between the renal arteries and the superior mesenteric artery, or sham operation (n = 16) was performed in pentobarbital sodium (60 mg/kg i.p.) anaesthesia as previously described^16^,^50^. After recovering from anaesthesia and on the first and second postoperative day, all animals received meloxicam (1.5 mg/kg p.o.) for postoperative analgesia.

### Experimental groups, chronic treatment protocol

5 days after the operations, sham and AAB animals were randomized into control or treatment groups (ShamCo, n = 8; ShamCin, n = 8; AABCo, n = 10; AABCin, n = 9). Treated animals received Cinaciguat (10 mg/kg p.o.) suspended in 0.5% methylcellulose solution via oral gavage, while control rats were given only the vehicle every day for 6 weeks. The dosage was adjusted to body weight, which was measured three times a week during the whole study period.

### Echocardiography

We performed echocardiographic measurements at the 3^rd^ and 6^th^ week after the operations as previously described^42^. Briefly, two-dimensional and M-mode echocardiographic images of long- and short (mid-papillary level)-axis were recorded in pentobarbital sodium (60 mg/kg i.p.) anaesthetised animals using a 13-MHz linear transducer (GE 12L-RS, GE Healthcare, Waukesha, WI, USA) connected to an echocardiographic imaging unit (Vivid i, GE Healthcare). Digital images (Fig. 1) were analysed by an investigator in blinded fashion using an image analysis software (EchoPac, GE Healthcare). LV anterior wall thickness (AWT), posterior wall thickness (PWT), LVEDD and LVESD in diastole (index: d) and systole (index: s) were measured on two-dimensional recordings of the short-axis at the mid-papillary muscle level. All values were averaged over three consecutive cycles. The following parameters were derived from these measurements: FS, end-diastolic (LVEDV) and end-systolic (LVESV) LV volumes, SV, EF, and LVM. To calculate LVMi, we normalized the LVM values to the body weight of the animal.

### Hemodynamic measurements: LV Pressure-Volume (P-V) analysis

P-V analysis was performed in each rat as previously described^51^. Briefly, rats were anesthetised with pentobarbital sodium (60 mg/kg i.p.), tracheotomised, intubated and ventilated. A polyethylene catheter was inserted into the left external jugular vein for fluid administration. A 2-Fr micro tip pressure-conductance catheter (SPR-838, Millar Instruments, Houston, TX, USA) was inserted into the right carotid artery and advanced into the ascending aorta, then the catheter was advanced into the LV under pressure control. LVESP and LVESV, LVEDP and LVEDV, dP/dt_max_, τ (according to the Glantz method), EF and SW were computed and calculated using a special P-V analysis program (PVAN, Millar Instruments). SV and CO were calculated and corrected according to *in vitro* and *in vivo* volume calibrations using PVAN software. In addition to the above parameters, the slope (E_es_) of the LV end-systolic P-V relationship (ESPVR; according to the parabolic curvilinear model^52^), PRSW, and dP/dt_max_-EDV were calculated as load-independent indices of LV contractility. At the end of each experiment, 100 μl of hypertonic saline was injected intravenously, and from the shift of P-V relations, parallel conductance volume was calculated by the software and used for the correction of the cardiac mass volume. The volume calibration of the conductance system was performed as previously described^42^. After completion of the hemodynamic measurements all animals were euthanized by exsanguination.

### Post mortem measurements

After euthanasia, the heart, the lung and the liver of the animals were immediately placed into cold saline and were measured on a scale. This was followed by the sampling of the organs, as described below. To exclude the natural variability between the weights of the animals, the right tibia of every rat was also prepared and its length measured^53^.

### Histology and immunohistochemistry

Hearts were harvested immediately after euthanasia, and samples were placed in 4% buffered paraformaldehyde solution. 5 μm thick heart sections were stained with haematoxylin and eosin, Picrosirius red, immunohistochemical staining for cGMP, and terminal deoxynucleotidyl transferase dUTP nick end labelling (TUNEL) staining to detect DNA strand breaks in LV myocardium. Light microscopic examination was performed with a Zeiss microscope (Axio Observer.Z1, Carl Zeiss, Jena, Germany), and digital images were captured using an imaging software (QCapture Pro 6.0, QImaging, Surrey, BC, Canada).

The mean value of transverse transnuclear widths of 100 randomly selected, longitudinally oriented LV cardiomyocytes represents each sample. The amount of myocardial collagen was determined by measuring the area fraction of the Picrosirius red-stained areas of five randomly selected visual fields (magnification: 200x) of subendocardial LV myocardium of each section with ImageJ software. Immunohistochemical reactivity for cGMP was examined with light microscopy at a magnification of 400x. Semi-quantitative scoring (scores 0–4; 0: no staining, 1: weak, 2: mild, 3: strong, 4: very strong staining) was performed by two people blinded to the groups as described elsewhere^54^. TUNEL positive cell nuclei were counted by two blinded observers in 10 fields of each section at 200x magnification. Data were normalized to the mean value of the ShamCo group and were used to perform statistical analysis.

### Biochemical measurements

After hemodynamic measurements were completed, blood samples from the inferior caval vein were collected in tubes rinsed with EDTA. The blood samples were centrifuged at 3,000 RPM for 15 min at 4 °C, then separated plasma was stored in aliquots at -80°C. Plasma level of cGMP was determined using an enzyme immunoassay kit as per manufacturer’s protocol (Amersham cGMP EIA Biotrak System, GE Healthcare, Little Chalfont, Buckinghamshire, UK).

### Cardiac mRNA analysis

LV myocardial tissue samples were harvested immediately after euthanasia, snap frozen in liquid nitrogen, and stored at −80 °C. LV tissue was homogenized in RLT buffer, and RNA was isolated from the ventricular samples using the RNeasy Fibrous Tissue Mini Kit (Qiagen, Hilden, Germany) according to the manufacturer’s instructions. Quantitative real-time PCR was performed with the StepOne-Plus Real-Time PCR System (Applied Biosystems, Foster City, CA, USA) in triplicates of each sample for the following targets: α- and β-isoform of myosin heavy chain (MHCα, MHCβ), endothelial nitric oxide synthase (NOS3), atrial natriuretic peptide (ANP), B cell lymphoma 2 (Bcl-2), 70 kDa heat shock protein (HSP70), sarcoplasmic and endoplasmic reticulum Ca^2+^-ATPase isoform 2a (SERCA2a) and phospholamban (Pln), all purchased from Applied Biosystems. Gene expression data were normalized to glyceraldehyde-3-phosphate dehydrogenase (GAPDH), and expression levels were calculated using the CT comparative method (2^−ΔCT^). All results are expressed as values normalized to a positive calibrator (a pool of cDNAs from all samples of the ShamCo group).

### Immunoblot analysis

Immunoblot analysis was performed as previously described^55^. Briefly, LV tissue samples were homogenized and were boiled with Laemmli buffer. Equal amounts of protein (30 μg) were loaded and separated on commercially available precast 4–12% SDS-PAGE gels (NuPAGE^®^ Novex^®^ Bis-Tris Mini Gel, Invitrogen, Carlsbad, CA, USA). Afterwards, proteins were transferred to nitrocellulose membrane by using a semi-dry electroblotting system (iBlot™ Gel Transfer Device, Invitrogen). Membranes were incubated overnight at 4 °C with primary antibodies (all purchased from Cell Signaling, Danvers, MA, USA, unless noted otherwise) against various target proteins as follows: members of NO signalling such as protein kinase G (PKG, primary antibody from Enzo Life Sciences, Plymouth Meeting, PA, USA), vasodilator-stimulated phosphoprotein (VASP) and phospho-VASP, phospholamban (Pln) and phospho-Pln as markers of PKG activity. After washing, membranes were incubated in horseradish peroxidase (HRP) – conjugated secondary antibody dilutions at room temperature (RT) for 1 h (anti-rabbit IgG or anti-mouse IgG as appropriate, 1:2000). Immunoblots were developed using Pierce^®^ ECL Western Blotting Substrate Kit (Thermo Scientific, Rockford, IL, USA). Protein band densities were quantified using GeneTools software (Syngene, Frederick, MD, USA). GAPDH (primary antibody from Millipore, Billerica, MA, USA) was used to assess equal protein loading. Values of protein band densities (after adjusting to GAPDH band densities) were normalized to the average value of the ShamCo group and were used to perform statistical analysis. Representative original immunoblots are shown in Supplementary Figure 1.

### Drugs

All drugs listed were purchased from Sigma-Aldrich (St. Louis, MO, USA) except for Cinaciguat, which is a kind gift of Bayer AG (Wuppertal, Germany).

### Statistical analysis

Statistical analysis was performed on a personal computer with a commercially available software (GraphPad Prism 6, La Jolla, CA, USA).

All data are expressed as mean ± standard error of the mean (SEM). After testing normal distribution of the data using the Shapiro-Wilk test, two-factorial analysis of variance (ANOVA) (with ‘aortic banding’ and ‘Cinaciguat treatment’ as factors) was carried out to detect independent effects of the factors (p_band_, p_treat_) and significant banding × treatment interactions (p_int_). Tukey’s *post hoc* testing was performed to evaluate differences between the groups. Data that did not show normal distribution were transformed logarithmically before performing two-factorial ANOVA.

A paired Student’s *t*-test was performed for comparing data of the echocardiographic measurements at 2 time points within a group. Differences were considered statistically significant when p < 0.05.

## Additional Information

**How to cite this article**: Németh, B. T. *et al.* Cinaciguat prevents the development of pathologic hypertrophy in a rat model of left ventricular pressure overload. *Sci. Rep.* **6**, 37166; doi: 10.1038/srep37166 (2016).

**Publisher’s note:** Springer Nature remains neutral with regard to jurisdictional claims in published maps and institutional affiliations.

## Supplementary Material

Supplementary Information

## Figures and Tables

**Table 1:** Echocardiographic measurements.

	3^rd^ week							6^th^ week						
	*ShamCo*	*ShamCin*	*AABCo*	*AABCin*	*p*_*band*_	*p*_*treat*_	*p*_*int*_	*ShamCo*	*ShamCin*	*AABCo*	*AABCin*	*p*_*band*_	*p*_*treat*_	*p*_*int*_
*AWT*_*d*_ *(mm)*	1.92 ± 0.04	1.85 ± 0.02	2.27 ± 0.05*	2.04 ± 0.05*#	<***0.0001***	***0.021***	***0.029***	2.03 ± 0.02	**1.97 ± 0.02&**	2.40 ± 0.04*&	2.21 ± 0.03*#**&**	<***0.0001***	***0.0004***	***0.010***
*AWT*_*s*_ *(mm)*	2.87 ± 0.09	3.00 ± 0.03	3.39 ± 0.08*	3.20 ± 0.05	<***0.0001***	*0.841*	***0.024***	3.09 ± 0.08	3.11 ± 0.04	3.49 ± 0.07*	3.33 ± 0.06*	<**0.0001**	*0.852*	***0.027***
*PWT*_*d*_ *(mm)*	1.78 ± 0.04	1.78 ± 0.04	2.15 ± 0.05*	**1.89 ± 0.06**#	<***0.0001***	***0.041***	***0.004***	1.85 ± 0.03	1.72 ± 0.03	2.32 ± 0.04*&	2.05 ± 0.07*#**&**	<***0.0001***	***0.001***	*0.112*
*PWT*_*s*_ *(mm)*	2.87 ± 0.12	2.94 ± 0.07	3.16 ± 0.09*	2.97 ± 0.04	***0.016***	*0.832*	***0.034***	2.80 ± 0.05	2.80 ± 0.04	3.29 ± 0.07*	3.03 ± 0.07*#	<***0.0001***	*0.127*	***0.014***
*LVEDD (mm)*	6.43 ± 0.08	6.35 ± 0.09	6.78 ± 0.18	6.54 ± 0.12	*0.066*	*0.234*	*0.537*	6.54 ± 0.11	6.65 ± 0.13	7.04 ± 0.15	**6.48 ± 0.18**#	*0.122*	*0.087*	***0.049***
*LVESD (mm)*	3.59 ± 0.08	3.30 ± 0.05	3.92 ± 0.20	3.60 ± 0.10	***0.021***	*0.050*	*0.898*	3.56 ± 0.12	**3.52 ± 0.12&**	4.19 ± 0.14*^&^	3.74 ± 0.12	***0.002***	*0.064*	*0.159*
*RWT (%)*	0.58 ± 0.01	0.57 ± 0.01	0.66 ± 0.02*	0.60 ± 0.02	***0.034***	*0.130*	*0.093*	0.59 ± 0.01	0.56 ± 0.02	0.67 ± 0.02*	0.64 ± 0.01*	<***0.0001***	*0.076*	*0.759*
*LVM (g)*	0.81 ± 0.03	0.76 ± 0.01	1.15 ± 0.06*	**0.90 ± 0.04**#	<***0.0001***	***0.003***	***0.020***	**0.88 ± 0.03&**	**0.85 ± 0.02&**	1.33 ± 0.05*&	**1.00 ± 0.04**#^&^	<***0.0001***	<***0.0001***	***0.001***
*LVMi (mg/g)*	2.35 ± 0.06	2.35 ± 0.03	3.38 ± 0.14*	**2.90 ± 0.12**#	<***0.0001***	*0.058*	***0.023***	**2.09 ± 0.05&**	**2.05 ± 0.05&**	3.15 ± 0.09*	2.57 ± 0.06*#**&**	<***0.0001***	***0.0002***	***0.001***
*FS (%)*	44 ± 2	47 ± 1	42 ± 1	44 ± 1	*0.180*	*0.061*	*0.518*	44 ± 1	46 ± 1	41 ± 1	43 ± 1	***0.009***	*0.128*	*0.821*
*EF (%)*	62 ± 2	66 ± 1	62 ± 1	69 ± 2*#	*0.310*	***0.002***	*0.339*	66 ± 2	69 ± 1	62 ± 1*	**69 ± 2**#	*0.232*	***0.013***	*0.339*

Indexes: d: diastole, s: systole; AWT: anterior wall thickness, PWT: posterior wall thickness; LVEDD: left ventricular end diastolic diameter, LVESD: left ventricular end systolic diameter; RWT: relative wall thickness; LVM: left ventricular mass, LVMi: left ventricular mass index; FS: fractional shortening; EF: ejection fraction; p_band_: *p* value of ‘aortic banding’ main effect; p_treat_: *p* value of ‘Cinaciguat treatment’ main effect; p_int_: interaction *p* value ^*^p<0.05 vs. ShamCo; ^#^p < 0.05 vs. AABCo; ^&^p < 0.05 vs. 3rd week.

**Table 2:** Baseline hemodynamic parameters.

	*ShamCo*	*ShamCin*	*AABCo*	*AABCin*	*pband*	*ptreat*	*pint*
*HR (1/min)*	433 ± 15	412 ± 17	435 ± 11	444 ± 17	*0.255*	*0.679*	*0.309*
*MAP (mmHg)*	134 ± 5	127 ± 5	183 ± 7*	177 ± 5*	<**0.0001**	*0.218*	*0.902*
*ESV (μl)*	103 ± 8	91 ± 8	97 ± 7	96 ± 8	*0.959*	*0.400*	*0.436*
*EDV (μl)*	221 ± 19	190 ± 13	233 ± 11	199 ± 16	*0.790*	*0.094*	*0.728*
*LVESP (mmHg)*	140 ± 4	138 ± 3	191 ± 14*	185 ± 6*	***<0.0001***	*0.610*	*0.903*
*LVEDP (mmHg)*	4.7 ± 0.5	4.1 ± 0.4	4.5 ± 0.3	4.9 ± 0.6	*0.248*	*0.741*	*0.484*
*SV (μl)*	145 ± 14	128 ± 7	156 ± 10	135 ± 7	*0.390*	*0.066*	*0.857*
*EF (%)*	60 ± 1	61 ± 2	61 ± 1	61 ± 1	*0.489*	*0.685*	*0.854*
*CO (ml/min)*	63.3 ± 6.7	52.4 ± 3.1	67.6 ± 4.6	56.5 ± 1.8	*0.373*	***0.025***	*0.984*
SW (mmHg*μl)	16.49 ± 1.76	14.29 ± 1.19	20.92 ± 1.64	17.67 ± 1.40	***0.017***	*0.088*	*0.736*
*E_a_ (mmHg/μl)*	1.03 ± 0.10	1.06 ± 0.07	1.28 ± 0.13	1.39 ± 0.07	***0.008***	*0.479*	*0.668*
*τ_G_ (ms)*	11.21 ± 0.49	11.33 ± 0.75	16.30 ± 1.65*	13.65 ± 0.66	***0.001***	*0.225*	*0.185*
*dP/dt_max_ (mmHg/s)*	8589 ± 460	8200 ± 543	10999 ± 430*	10374 ± 648	***0.0001***	*0.338*	*0.822*

HR: heart rate; ESV: end-systolic volume, EDV: end diastolic volume; LVSP: left ventricular systolic pressure, LVEDP: left ventricular end diastolic pressure; MAP: mean arterial pressure; SV: stroke volume; EF: ejection fraction; CO: cardiac output; SW: stroke work; E_a_: arterial elastance; τ_G_: time constant of active relaxation according to Glantz (tau); dP/dt**max**: maximum rate of pressure change; p_band_: *p* value of ‘aortic banding’ main effect; p_treat_: *p* value of ‘Cinaciguat treatment’ main effect; p_int_: interaction *p* value ^*^p < 0.05 vs. ShamCo; ^#^p < 0.05 vs. AABCo.
