# Supplementary material for: Cinaciguat prevents the development of pathologic hypertrophy in a rat model of left ventricular pressure overload
Source: Sci Rep. 2016 Nov 17;6:37166. doi: 10.1038/srep37166 (PMC5112572; doi:10.1038/srep37166)
Supplement: Supplementary Information [file srep37166-s1.pdf]

Supplementary material

Cinaciguat prevents the development of pathologic hypertrophy in a rat model of left ventricular pressure overload

Balázs Tamás Németh MD[a], Csaba Mátyás MD[a], Attila Oláh MD PhD[a], Árpád Lux MD[a], László Hidi MD[a], Mihály Ruppert MD[a], Dalma Kellermayer MD[a], Gábor Kökény MD PhD[b], Gábor Szabó MD PhD[c], Béla Merkely MD PhD[a#], Tamás Radovits MD PhD[a*#]

[a]Heart and Vascular Center, Semmelweis University, Városmajor u. 68., 1122 Budapest, Hungary

[b]Institute of Pathophysiology, Semmelweis University, Nagyvárad tér 4., 1089 Budapest, Hungary

[c]Department of Cardiac Surgery, University of Heidelberg, Im Neuenheimer Feld 110., 69210 Heidelberg, Germany

[#]T. Radovits and B. Merkely contributed equally to this work.

Corresponding author:

Tamás Radovits MD, PhD

Experimental Research Laboratory

Heart and Vascular Center, Semmelweis University

1122 Budapest, Városmajor u. 68.

Hungary

Tel.: +36-1-458-6810, Fax: +36-1-458-6842

Email: radovitstamas@yahoo.com

Methods

All animals received humane care in compliance with the “Principles of Laboratory Animal Care”, formulated by the National Society for Medical Research and the Guide for the Care and Use of Laboratory Animals, prepared by the Institute of Laboratory Animal Resources and published by the National Institutes of Health (NIH Publication No. 85-23, Revised 1996). All procedures and handling of the animals during the study were reviewed and approved by the Ethical Committee of Hungary for Animal Experimentation. Young adult (10 week old, body weight = 220–240g) male Wistar rats (n=35) ("Toxi-Coop" Zrt., Dunakeszi, Hungary) were housed in a room with constant temperature of 22±2°C with a 12h light-dark cycle, were fed a standard laboratory rat chow ad libitum and had free access to water.

2.1. Abdominal aortic banding procedure

After acclimation, banding of the abdominal aorta (AAB, n=19) between the renal arteries and the superior mesenteric artery, or sham operation (n=16) was performed in pentobarbital sodium (60mg/kg i.p.) anaesthesia as previously described[1]. Briefly, to achieve a standard degree of stenosis in our aortic banded animals, a blunted needle with an outer diameter of 20 Gauge (G, 0.9mm) was placed in parallel with the aorta, around which a 2-0 surgical suture was tightened in all cases. Then, the needle was removed, leaving a standard 20G stenosis behind. Sham operation comprised all steps excluding the banding procedure, which was replaced by a 1 min occlusion at the same level of the aorta. After recovering from anaesthesia and on the first and second postoperative day, all animals received meloxicam (1.5mg/kg p.o.) for postoperative analgesia.

2.2. Experimental groups, chronic treatment protocol

5 days after the operations, sham and AAB animals were randomized into control or treatment groups (ShamCo, n=8; ShamCin, n=8; AABCo, n=10; AABCin, n=9). Treated animals received Cinaciguat (10mg/kg p.o.) suspended in 0.5% methylcellulose solution via oral gavage, while control rats were given only the vehicle every day for 6 weeks. The dosage was adjusted to body weight, which was measured three times a week during the whole study period.

2.3. Echocardiography

We performed echocardiographic measurements at the 3rd and 6th week after the operations as previously described[2]. Briefly, the rats were anesthetised with pentobarbital sodium (60mg/kg i.p.), were placed on controlled heating pads, and the core temperature, measured via rectal probe, was maintained at 37°C. After the anterior chest was shaved, transthoracic echocardiography was performed in the supine position by an investigator blinded to the experimental groups. Two-dimensional and M-mode echocardiographic images of long- and short (mid-papillary level)-axis were recorded using a 13-MHz linear transducer (GE 12L-RS, GE Healthcare, Waukesha, WI, USA) connected to an echocardiographic imaging unit (Vivid i, GE Healthcare). Digital images were analysed by an investigator in blinded fashion using an image analysis software (EchoPac, GE Healthcare). On two-dimensional recordings of the short-axis at the mid-papillary muscle level, left ventricular (LV) anterior wall thickness (AWT), posterior wall thickness (PWT) in diastole (index: d) and systole (index: s), and LV end-diastolic (LVEDD) and end-systolic (LVESD) diameters were measured. In addition, end-diastolic and end-systolic LV areas were planimetered from short- and long-axis two-dimensional recordings. End systole was defined as the time point of minimal LV dimensions, and end diastole as the time point of maximal dimensions. All values were averaged over three consecutive cycles. The following parameters were derived from these measurements: fractional shortening (FS) was calculated as [(LVEDD-LVESD)/LVEDD]*100. End-diastolic

(LVEDV) and end-systolic (LVESV) LV volumes were estimated according to a validated geometrical model, the biplane ellipsoid model[3]. Stroke volume (SV) was calculated as LVEDV-LVESV. Ejection fraction (EF) was determined as (SV/LVEDV)*100. LV mass (LVM) was calculated according to a cubic formula, suggested by Devereux et al.[4]: LV mass = $([(LVEDD+AWTd+PWTd)^3-LVEDD^3]*1.04)*0.8+0.14$. To calculate LVM index (LVMi), we normalized the LVM values to the body weight of the animal.

2.4. Hemodynamic Measurements: LV Pressure-Volume (P-V) Analysis

Rats were anesthetised with pentobarbital sodium (60mg/kg i.p.), tracheotomised, intubated and ventilated with a tidal volume and frequency adjusted to body weight using an Inspira Advanced Safety Ventilator (MA1 557058, Harvard Apparatus, Holliston, MA, USA). Animals were placed on controlled heating pads, and the core temperature, measured via rectal probe, was maintained at 37°C. A polyethylene catheter was inserted into the left external jugular vein for fluid administration. A 2-Fr micro tip pressure-conductance catheter (SPR-838, Millar Instruments, Houston, TX, USA) was inserted into the right carotid artery and advanced into the ascending aorta. After stabilization for 5 min, arterial blood pressure and heart rate (HR) were recorded. Then, the catheter was advanced into the LV under pressure control. After stabilization for 5 min, signals were continuously recorded at a sampling rate of 1,000 samples/s using a P-V conductance system (MPVS-Ultra, Millar Instruments) connected to the PowerLab 16/30 data acquisition system (AD Instruments, Colorado Springs, CO, USA), stored, and displayed on a personal computer by the LabChart5 Software System (AD Instruments). LV end-systolic pressure and volume (LVESP and LVESV), LV end-diastolic pressure and volume (LVEDP and LVEDV), the maximum (dP/dt_{max}) rate of LV pressure change, time constant of LV pressure decay [τ; according to the Glantz method], EF and stroke work (SW) were computed and calculated using a special P-V analysis program (PVAN, Millar Instruments). SV and cardiac output (CO) were calculated

and corrected according to in vitro and in vivo volume calibrations using PVAN software. In addition to the above parameters, P-V loops recorded at different preloads can be used to derive other useful systolic function indexes that are less influenced by loading conditions and cardiac mass[5]. Therefore, LV P-V relations were measured by transiently reducing preload by compressing the inferior caval vein under the diaphragm with a cotton-tipped applicator. The slope [end-systolic elastance (E_{es})] of the LV end-systolic P-V relationship (ESPVR; according to the parabolic curvilinear model[6]), preload recruitable SW (PRSW), and the slope of the dP/dt_{max}-end diastolic volume relationship (dP/dt_{max}-EDV) were calculated as load-independent indices of LV contractility. At the end of each experiment, 100 μl of hypertonic saline was injected intravenously, and from the shift of P-V relations, parallel conductance volume was calculated by the software and used for the correction of the cardiac mass volume. The volume calibration of the conductance system was performed as previously described[2]. Briefly, nine cylindrical holes in a block 1 cm deep and with known diameters ranging from 2 to 11 mm were filled with fresh heparinized whole rat blood. In this calibration, the linear volume-conductance regression of the absolute volume in each cylinder versus the raw signal acquired by the conductance catheter was used as the volume calibration formula. After completion of the hemodynamic measurements all animals were euthanized by exsanguination.

2.5. Post mortem measurements

After euthanasia, the heart, the lung and the liver of the animals were immediately placed into cold saline and were measured on a scale. This was followed by the sampling of the organs, as described below. To exclude the natural variability between the weights of the animals, the right tibia of every rat was also prepared and its length measured[7].

2.6. Histology and immunohistochemistry

Hearts were harvested immediately after euthanasia, and samples were placed in 4% buffered paraformaldehyde solution. Transverse transmural slices of the ventricles were sectioned (5 μm) and conventionally processed for histological examination. Heart sections were stained with hematoxylin and eosin and Picrosirius red. Light microscopic examination was performed with a Zeiss microscope (Axio Observer.Z1, Carl Zeiss, Jena, Germany), and digital images were captured using an imaging software (QCapture Pro 6.0, QImaging, Surrey, BC, Canada). The transverse transnuclear widths of randomly selected, longitudinally oriented cardiomyocytes were measured by a single investigator after calibrating the system. The mean value of 100 LV cardiomyocytes represents each sample. The amount of myocardial collagen was determined by measuring the area fraction of the Picrosirius red-stained areas of the sections with ImageJ software. The mean value of five randomly selected visual fields (magnification, 200x) of free LV wall represents each sample.

According to the method previously described[7], immunohistochemical analysis for cGMP was performed on paraffin sections of the heart by using the avidin-biotin method (anti-cGMP mouse monoclonal antibody 1:2000, Abcam, Cambridge, UK). Immunohistochemical reactivity was examined with light microscopy at a magnification of 400x. Semiquantitative scoring (scores 0–4; 0: no staining, 1: weak, 2: mild, 3: strong, 4: very strong staining) was performed by two people blinded to the groups as described elsewhere[8].

2.7. Terminal deoxynucleotidyl transferase dUTP nick end labeling (TUNEL) assay

Paraffin embedded, 5 μm thick heart tissue sections were used to detect DNA strand breaks in LV myocardium. TUNEL assay was performed using a commercially available kit (DeadEnd™ Colorimetric TUNEL System, Promega, Mannheim, Germany) according to the manufacturer's protocol. TUNEL positive cell nuclei were counted by two blinded observers in 10 fields of each section at 200x magnification. Data were normalized to the mean value of the ShamCo group and were used to perform statistical analysis.

2.8. Biochemical Measurements

After hemodynamic measurements were completed, blood samples from the inferior caval vein were collected in tubes prerinsed with EDTA. The blood samples were centrifuged at 3,000 RPM for 15 min at 4°C, then separated plasma was stored in aliquots at -80°C. Plasma level of cGMP was determined using an enzyme immunoassay kit as per manufacturer's protocol (Amersham cGMP EIA Biotrak System, GE Healthcare, Little Chalfont, Buckinghamshire, UK).

2.9. Cardiac mRNA Analysis

LV myocardial tissue samples were harvested immediately after euthanasia, snap frozen in liquid nitrogen, and stored at -80°C. LV tissue was homogenized in RLT buffer, and RNA was isolated from the ventricular samples using the RNeasy Fibrous Tissue Mini Kit (Qiagen, Hilden, Germany) according to the manufacturer's instructions and quantified by measuring optical density at 260 nm. RNA purity was ensured by obtaining a 260/280 nm optical density ratio of >2.0. Reverse transcription reaction (1μg total RNA of each sample) was completed using the QuantiTect Reverse Transcription Kit (Qiagen). Quantitative real-time PCR was performed with the StepOne-Plus Real-Time PCR System (Applied Biosystems, Foster City, CA, USA) in triplicates of each sample in a volume of 10μl in each well containing cDNA (1μl), TaqMan Universal PCR MasterMix (5μl), and a TaqMan Gene Expression Assay for the following targets (0.5μl, Suppl. Table 1): α- and β-isoform of myosin heavy chain (α-MHC, β-MHC), endothelial nitric oxide synthase (NOS3), atrial natriuretic peptide (ANP), B cell lymphoma 2 (Bcl-2), 70 kDa heat shock protein (HSP70), sarcoplasmic and endoplasmic reticulum Ca^{2+}-ATPase isoform 2a (SERCA2a) and phospholamban (Pln), all purchased from Applied Biosystems. Gene expression data were normalized to glyceraldehyde-3-phosphate dehydrogenase (GAPDH; reference gene), and expression levels were calculated using the CT

comparative method ($2^{-\Delta CT}$). All results are expressed as values normalized to a positive calibrator (a pool of cDNAs from all samples of the ShamCo group).

2.10. Immunoblot analysis

LV tissue samples were homogenized in radioimmunoprecipitation assay lysis buffer (RIPA; 50mM Tris HCl pH 8, 150mM NaCl, 1% NP-40, 0.5% sodium deoxycholate, 0.1% SDS) containing Complete Protease Inhibitor Cocktail (Roche, Mannheim, Germany). Protein concentration was determined using the Pierce® BCA Protein Assay Kit (Thermo Scientific, Rockford, IL, USA). Samples were mixed with 2× Laemmli buffer and boiled at 95ºC for 5 min. Equal amounts of protein (30 µg) were loaded and separated on commercially available precast 4–12% SDS-PAGE gels (NuPAGE® Novex® Bis-Tris Mini Gel, Invitrogen, Carlsbad, CA, USA). Afterwards, proteins were transferred to nitrocellulose membrane by using a semi-dry electroblotting system (iBlot™ Gel Transfer Device, Invitrogen). Membranes were blocked in 5% non-fat milk in Tris-buffered saline containing 0.1% Tween-20 (TTBS) for 1 h. After blocking, membranes were incubated overnight at 4ºC with primary antibodies (diluted in 1% bovine serum albumin in TTBS, all purchased from Cell Signaling, Danvers, MA, USA, unless noted otherwise) against various target proteins as follows (Suppl. Table 2): members of NO signalling such as protein kinase G (PKG), vasodilator-stimulated phosphoprotein (VASP) and phospho-VASP, phospholamban (Pln) and phospho-Pln as markers of PKG activity. After washing, membranes were incubated in horseradish peroxidase (HRP) – conjugated secondary antibody dilutions at room temperature (RT) for 1 h (anti-rabbit IgG or anti-mouse IgG as appropriate, 1:2000, Cell Signaling). Immunoblots were developed using Pierce® ECL Western Blotting Substrate Kit (Thermo Scientific). Protein band densities were quantified using GeneTools software (Syngene, Frederick, MD, USA). GAPDH (primary antibody purchased from Millipore, Billerica, MA, USA) was used to assess equal protein loading. Values of protein band densities (after adjusting to GAPDH

band densities) were normalized to the average value of the ShamCo group and were used to perform statistical analysis. Representative original immunoblots are shown in Supplementary Figure 1.

2.11. Drugs

All drugs listed were purchased from Sigma-Aldrich (St. Louis, MO, USA) except for Cinaciguat, which is a kind gift of Bayer AG (Leverkusen, Germany).

2.12. Statistical Analysis

Statistical analysis was performed on a personal computer with a commercially available software (GraphPad Prism 6, La Jolla, CA, USA). All data are expressed as means ± standard error of the mean. After testing normal distribution of the data using the Shapiro-Wilk test, two-factorial analysis of variance (ANOVA) (with 'aortic banding' and 'Cinaciguat treatment' as factors) was carried out to detect independent effects of the factors (p_{band}, p_{treat}) and significant banding×treatment interactions (p_{int}). Tukey's *post hoc* testing was performed to evaluate differences between the groups. Data that did not show normal distribution were transformed logarithmically before performing two-factorial ANOVA. A paired Student's t-test was performed for comparing data of the echocardiographic measurements at 2 time points within a group. Differences were considered statistically significant when $p<0.05$.

Supplementary Tables and Figure

Supplementary Table 1. – TaqMan® Gene Expression Assays used for qRT-PCR

Target gene	*Abbreviation*	*Assay ID*
natriuretic peptide A	ANP	Rn00561661_m1
myosin heavy chain alfa	α-MHC	Rn00568304_m1
myosin heavy chain beta	β-MHC	Rn00568328_m1
B-cell CLL/lymphoma 2	Bcl-2	Rn99999125_m1
endothelial nitric oxide synthase	NOS3	Rn02132634_s1
heat shock 70kD protein 1A	HSP70	Rn04224718_u1
phospholamban	Pln	Rn01434045_m1
sarcoplasmic and endoplasmic reticulum Ca^{2+}-ATPase isoform 2a	SERCA2a	Rn00568762_m1
glyceraldehyde-3-phosphate dehydrogenase	GAPDH	Rn01775763_g1

Supplementary Table 2. – Antibodies used for immunoblot analysis

Target protein	*Abbreviation*	*Primary antibody*	*Dilution*	*Molecular mass*
protein kinase G	PKG	ADI-KAP-PK005-F (Enzo Life Sciences, Plymouth Meeting, PA, USA)	1:1000	75 kDa
vasodilator-stimulated phosphoprotein	VASP	3112 (Cell Signaling, Danvers, MA, USA)	1:1000	50 kDa
phospho-VASP	p-VASP	3114 (Cell Signaling)	1:500	50 kDa
phospholamban	Pln	8495 (Cell Signaling)	1:5000	24 kDa
phospho-Pln	p-Pln	8496 (Cell Signaling)	1:5000	24 kDa
glyceraldehyde-3-phosphate dehydrogenase	GAPDH	MAB374 (Millipore, Billerica, MA, USA)	1:10000	38 kDa

The table shows the primary antibodies (against various protein targets and reference protein glyceraldehyde-3-phosphate dehydrogenase) used in Western blot experiments.

Supplementary Table 3. – Organ weights

	ShamCo	*ShamCin*	*AABCo*	*AABCin*	p_{band}	p_{treat}	p_{int}
HW (g)	1.29±0.03	1.21±0.03	1.65±0.07*	1.47±0.05*	***<0.0001***	***0.019***	*0.346*
LuW (g)	1.72±0.05	1.70±0.06	1.88±0.03	1.81±0.09	***0.029***	*0.493*	*0.708*
LiW (g)	12.72±0.70	13.39±0.61	13.38±0.48	12.06±0.74	*0.832*	*0.878*	***0.011***
HW/BW (mg/g)	3.07±0.10	2.91±0.08	3.81±0.16*	3.66±0.12*	***<0.0001***	*0.207*	*0.941*
HW/TL (mg/mm)	29.3±0.8	28.1±0.9	38.4±1.5*	33.5±0.7*#	***<0.0001***	***0.008***	*0.094*
LuW/TL (mg/mm)	39.2±1.1	39.5±1.3	43.9±0.9*	41.9±1.8	***0.007***	*0.733*	*0.278*
LiW/TL (mg/mm)	289.5±14.3	312.0±15.7	311.7±9.2	278.7±15.6#	*0.627*	*0.645*	***0.002***

HW: heart weight; LuW: lung weight; LiW: liver weight; /BW: normalization to body weight; /TL: normalization to tibial length; p_{band}: *p* value of ‘aortic banding’ main effect; p_{treat}: *p* value of ‘Cinaciguat treatment’ main effect; p_{int}: interaction *p* value

*: p<0.05 vs. ShamCo; #: p<0.05 vs. AABCo

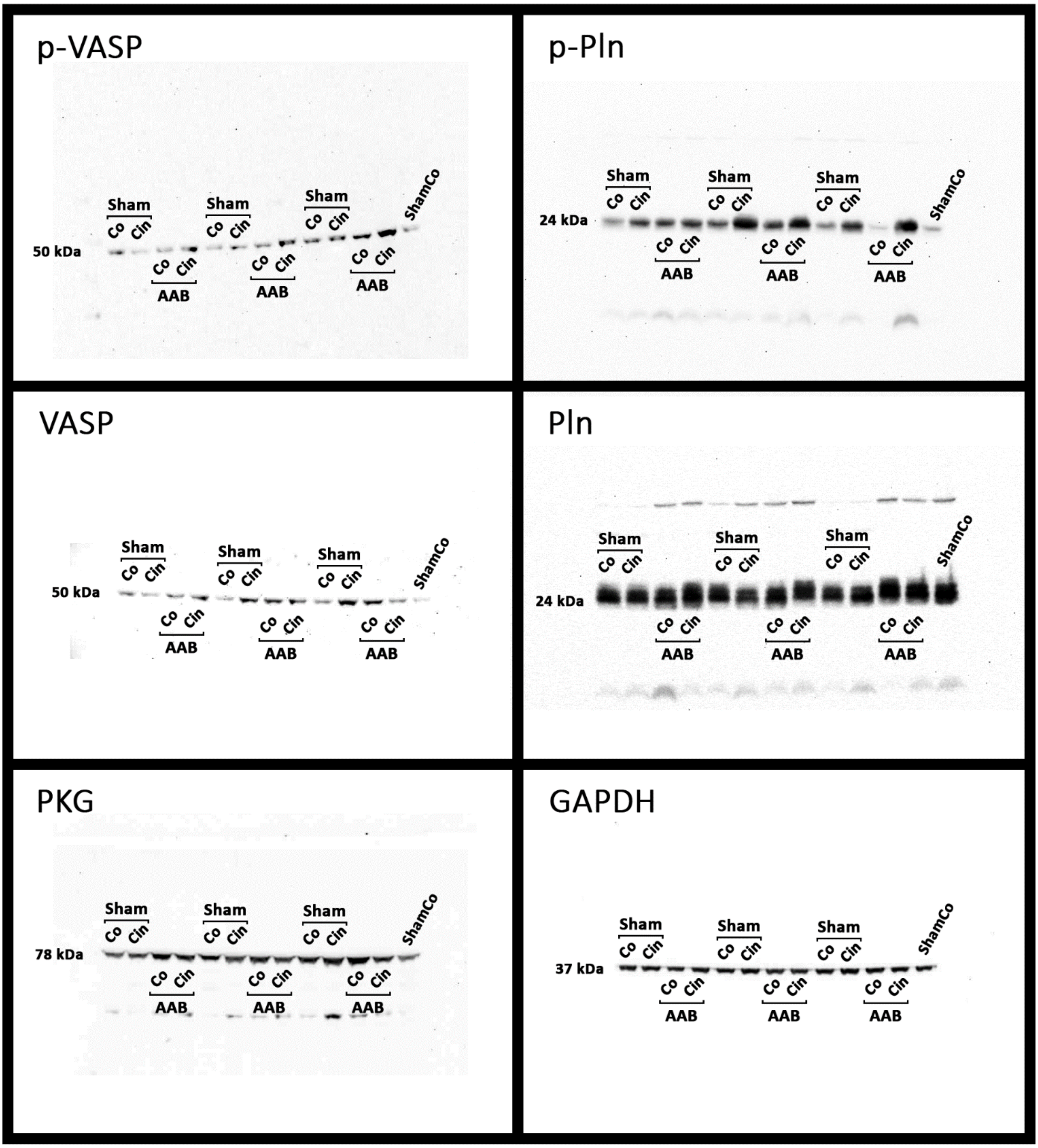

Supplementary Figure 1. Representative immunoblots showing antibody specificity

The last ShamCo band on every blot was used to normalize differences in chemiluminescence between blots incubated with the same antibody.

GAPDH: glyceraldehyde 3-phosphate dehydrogenase; PKG: protein kinase G; Pln: phospholamban; p-Pln: phospho-Pln; VASP: vasodilator stimulated phosphoprotein; p-VASP: phospho-VASP

References

1. Gao S, Long CL, Wang RH, Wang H. K(ATP) activation prevents progression of cardiac hypertrophy to failure induced by pressure overload via protecting endothelial function. *Cardiovasc Res* **83**, 444-456 (2009).

2. Radovits T, Olah A, Lux A, Nemeth BT, Hidi L, Birtalan E, Kellermayer D, Matyas C, Szabo G, Merkely B. Rat model of exercise-induced cardiac hypertrophy: hemodynamic characterization using left ventricular pressure-volume analysis. *Am J Physiol Heart Circ Physiol* **305**, H124-134 (2013).

3. van de Weijer T, van Ewijk PA, Zandbergen HR, Slenter JM, Kessels AG, Wildberger JE, Hesselink MKC, Schrauwen P, Schrauwen-Hinderling VB, Kooi ME. Geometrical models for cardiac MRI in rodents: comparison of quantification of left ventricular volumes and function by various geometrical models with a full-volume MRI data set in rodents. *Am J Physiol Heart Circ Physiol* **302,** H709-H715 (2012).

4. Devereux RB, Alonso DR, Lutas EM, Gottlieb GJ, Campo E, Sachs I, Reichek N. Echocardiographic assessment of left ventricular hypertrophy: comparison to necropsy findings. *Am J Cardiol* **57**, 450-458 (1986).

5. Oláh A, Németh BT, Mátyás C, Horváth EM, Hidi L, Birtalan E, Kellermayer D, Ruppert M, Merkely G, Szabó G, Merkely B, Radovits T. Cardiac effects of acute exhaustive exercise in a rat model. *Int J Cardiol* 2015;**182**:258-266.

6. Kass DA, Beyar R, Lankford E, Heard M, Maughan WL, Sagawa K. Influence of contractile state on curvilinearity of in situ end-systolic pressure-volume relations. *Circulation* **79,** 167-178 (1989).

7. Yin FC, Spurgeon HA, Rakusan K, Weisfeldt ML, Lakatta EG. Use of tibial length to quantify cardiac hypertrophy: application in the aging rat. *Am J Physiol* **243**, H941-947 (1982).

8. Fang L, Radovits T, Szabo G, Mozes MM, Rosivall L, Kokeny G. Selective phosphodiesterase-5 (PDE-5) inhibitor vardenafil ameliorates renal damage in type 1 diabetic rats by restoring cyclic 3',5' guanosine monophosphate (cGMP) level in podocytes. *Nephrol Dial Transplant* **28**, 1751-1761 (2013).

9. Radovits T, Bomicke T, Kokeny G, Arif R, Loganathan S, Kecsan K, Korkmaz S, Barnucz E, Sandner P, Karck M, Szabo G. The phosphodiesterase-5 inhibitor vardenafil improves cardiovascular dysfunction in experimental diabetes mellitus. *Br J Pharmacol* **156**, 909-919 (2009).
